# Effectiveness of Gel‐immersion Endoscopic Injection Sclerotherapy Under Texture and Color Enhancement Imaging for Esophageal Varices: A Comparison of Variceal Visibility Under Gel With White Light Imaging

**DOI:** 10.1002/deo2.70201

**Published:** 2025-09-22

**Authors:** Tsunetaka Kato, Takuto Hikichi, Jun Nakamura, Minami Hashimoto, Takumi Yanagita, Mitsuru Otsuka, Daiki Nemoto, Rei Suzuki, Mitsuru Sugimoto, Hiroyuki Asama, Kentaro Sato, Hiroshi Shimizu, Kento Osawa, Rei Ohira, Masao Kobayakawa, Hiromasa Ohira

**Affiliations:** ^1^ Department of Endoscopy Fukushima Medical University Hospital Fukushima Japan; ^2^ Department of Gastroenterology Fukushima Medical University School of Medicine Fukushima Japan; ^3^ Medical Research Center Fukushima Medical University Fukushima Japan

**Keywords:** endoscopic injection sclerotherapy, esophageal varices, gel‐immersion endoscopy, luminance gradient, texture and color enhancement imaging

## Abstract

**Objective:**

Gel‐immersion endoscopic injection sclerotherapy (GI‐EIS) addresses the technical challenges in intravariceal injection for esophageal varices (EVs). However, gel accumulation may obscure the variceal morphology. Thus, we developed GI‐EIS under texture and color enhancement imaging (TXI) and evaluated its effectiveness.

**Methods:**

This study included EV patients who underwent primary prophylactic intravariceal EIS. Patients were divided into GI‐EIS under TXI and conventional EIS groups. Primary outcomes were the success rates of intravariceal sclerosant injection and sclerosant injection into the afferent vessels. Secondary outcomes included the visibility score of EV morphology under TXI compared with white light imaging (WLI) during gel‐immersion and luminance gradient across the EVs.

**Results:**

Overall, 32 patients (16 in each group) were evaluated. The success rate of intravariceal sclerosant injection was comparable between GI‐EIS under TXI and conventional EIS (93.8% vs. 87.5%, *p* = 0.54). However, injection into the afferent vessels was significantly more successful with GI‐EIS under TXI (87.5% vs. 43.8%, *p* < 0.01). The visibility score of the variceal morphology under TXI was consistently five points in all cases. The luminance gradient was significantly higher under TXI than under WLI (TXI vs. WLI; 0.95 vs. 0.68; *p* < 0.01).

**Conclusion:**

GI‐EIS under TXI provided improved visualization of variceal morphology and enhanced success of injection into afferent vessels, suggesting that TXI may optimize the therapeutic performance of GI‐EIS for EV.

## 1 Introduction

Patients with persistent portal hypertension secondary to liver cirrhosis frequently develop esophageal varices (EVs) [1]. EV rupture can cause life‐threatening complications, including massive hemorrhage and hepatic failure [2, 3], making primary prophylactic treatment crucial for patients with high bleeding risk.

Endoscopic injection sclerotherapy (EIS), an effective modality for EVs, is associated with a lower recurrence rate compared to other treatments [4, 5, 6, 7]. However, EIS is more technically demanding than endoscopic variceal ligation (EVL) [8, 9]. The technical difficulty of conventional EIS is attributed to gas insufflation, which reduces the diameter of the varices along the axis of the needle puncture and stretches the esophageal wall, frequently inducing peristalsis [10]. To address these limitations, gel‐immersion EIS (GI‐EIS) was developed by adapting gel‐immersion endoscopy [11, 12, 13] which maintains luminal distension using gel instead of gas, to EIS procedures [14, 15, 16].

GI‐EIS provides several advantages. First, the gel reduces esophageal wall tension and suppresses peristalsis while preserving the variceal diameter, thus facilitating stable sclerosant injection. Second, gel accumulation within the esophageal lumen ensures a consistently clear endoscopic field. However, GI‐EIS has certain limitations as well. When the esophageal lumen is filled with gel and degassed, the esophageal mucosa relaxes, and the mucosa surrounding the EVs becomes elevated [17]. Consequently, the EV morphology becomes relatively indistinct, making visualization more challenging compared to gas insufflation. In some cases, the optimal site for puncture could not be clearly identified owing to this limitation.

Texture and color enhancement imaging (TXI; Olympus Medical Systems Corporation, Tokyo, Japan), a novel image‐enhanced endoscopy modality, has been recently developed. It enhances texture, brightness, and color, improving gastrointestinal lesion visibility. TXI has demonstrated utility in detecting early‐stage gastric cancer and gastritis [18]. We have observed that compared with conventional white light imaging (WLI), TXI enhances variceal morphology visibility during GI‐EIS, and we have designated this technique as “GI‐EIS under TXI” [19]. Accordingly, we aimed to evaluate the effectiveness of GI‐EIS under TXI.

## 2 Methods

### 2.1 Study Design and Patients

This retrospective observational study was conducted at the Fukushima Medical University Hospital from April 2022 to December 2024. From April 2022 to April 2023, only conventional EIS was performed, and from September 2023 to December 2024, only GI‐EIS under TXI was performed. Given that multiple treatment methods were implemented from May 2023 to September 2023, cases from this period were excluded. This study was conducted in accordance with the guidelines stipulated in the Declaration of Helsinki and was approved by the Ethics Committee of Fukushima Medical University (approval No. 2406).

The study eligibility criteria included consecutive EV patients undergoing primary prophylactic intravariceal EIS as an initial treatment between the two periods. The clinical indication for primary prophylactic EIS followed the criteria outlined in *The General Rules for the Study of Portal Hypertension* by the Japan Society for Portal Hypertension. Specifically, treatment was indicated for EVs classified as ≥F2 in morphology or those exhibiting red color signs, based on the endoscopic classification criteria [20]. Moreover, clinical and procedural data were retrospectively collected from the electronic medical records and institutional endoscopy database.

### 2.2 Clinical Procedure of Conventional EIS or GI‐EIS Under TXI

All procedures were performed under X‐ray fluoroscopic guidance with intravenous anesthesia using propofol [21] by two expert endoscopists certified by the Japan Gastroenterological Endoscopy Society (Tsunetaka Kato and Takumi Yanagita). The EVIS X1 endoscopic system (Olympus Medical Systems Corporation, Tokyo, Japan) and a therapeutic endoscope (GIF‐H290T; Olympus Medical Systems Corporation) were used in all procedures. The WLI settings were standardized to structural enhancement level B8 and color enhancement level 0. TXI Mode 1 was exclusively used for the TXI observations. All endoscopic images were archived using the Solemio ENDO system (Olympus Medical Systems Corporation).

**FIGURE 1:**
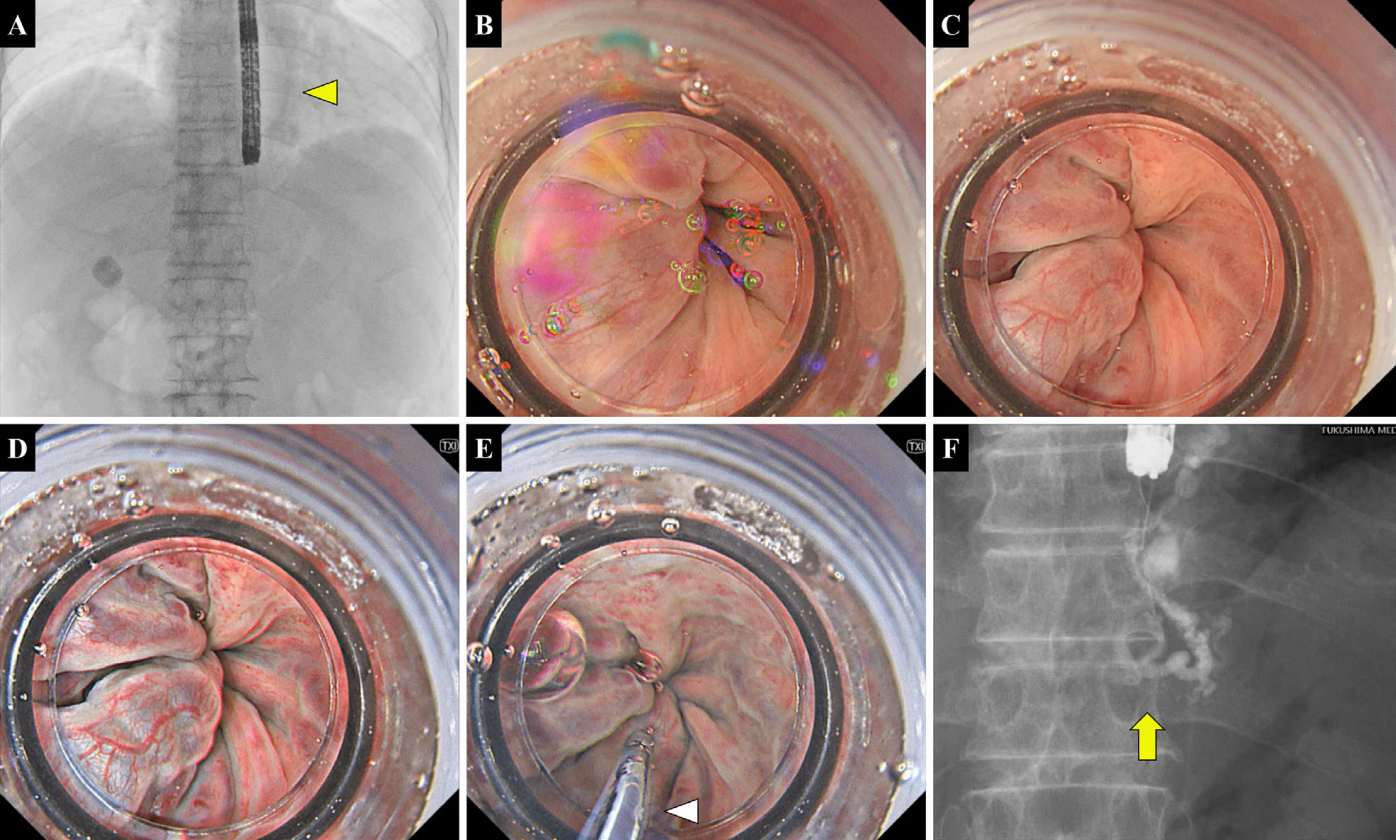
GI‐EIS procedure under TXI: (A) Following adequate deflation of the gastric gas, the EIS balloon attached to the endoscope is inflated with air (yellow arrowhead). (B) Gel is injected into the esophageal lumen via the endoscope's working channel. (C) WLI image of the gel‐filled esophageal lumen. (D) TXI image of the gel‐filled esophageal lumen. (E) EV is punctured under TXI guidance, and blood reflux into the catheter is confirmed (white arrowhead). (F) Ethanolamine oleate is injected under fluoroscopic guidance, and its progression into the afferent vessels is visualized (yellow arrow). EIS, endoscopic injection sclerotherapy; EV, esophageal varix; GI‐EIS, gel‐immersion endoscopic injection sclerotherapy; TXI, texture and color enhancement imaging; WLI, white light imaging.

For conventional EIS, an endoscope equipped with a transparent hood compatible with EVL (air‐pressure‐driven EVL device; SB Kawakami Co., Ltd., Kanagawa, Japan) and an EIS balloon (Create Medic Co., Ltd., Kanagawa, Japan) was inserted into the esophagus. After identifying the EVs under WLI, approximately 25 mL of air was injected into the balloon attached to the endoscope to block the EV blood flow (Figure 1A). A 5% ethanolamine oleate (EO) solution (Oldamin; Asuka Pharmaceutical Co., Ltd., Tokyo, Japan) diluted with a contrast medium was prefilled into a puncture needle (Varixer Type C; TOP Co., Ltd., Tokyo, Japan), and the EV was punctured under WLI guidance and CO_2_ insufflation.

Conversely, in the GI‐EIS under TXI, air was injected into the balloon to not only block the blood flow of the EVs but also prevent gel reflux into the oral cavity. Subsequently, a viscous gel (Viscoclear; Otsuka Pharmaceutical Co., Ltd., Tokushima, Japan) was introduced into the esophageal lumen via the working channel (Figure 1B). By thoroughly aspirating the gastric gas with the endoscope before gel injection, the gel tends to accumulate more easily in the esophageal lumen. After the lumen was adequately filled with gel, a puncture needle prefilled with a 5% mixture of EO diluted with a contrast medium was inserted. Additional gel was administered as needed through the water jet channel. The endoscopic view was subsequently switched from WLI (Figure 1C) to TXI Mode 1 (Figure 1D), and the EV was punctured under TXI guidance.

After puncturing into the EV, in both procedures, retrograde blood flow into the catheter was confirmed (Figure 1E), and the sclerosant was injected under fluoroscopic guidance (Figure 1F). Injection continued until the sclerosant reached the afferent vessels. When feasible, the needle was left in place for 5–10 min before removal to ensure adequate sclerosis. Finally, the balloon was deflated, and one puncture was completed. When post‐puncture bleeding persisted or preventive hemostasis was considered necessary, EVL was performed at the puncture site.

### 2.3 Evaluated Outcomes

The following treatment outcomes were evaluated: (1) success rate of intravariceal sclerosant injection; (2) success rate of sclerosant injection into the afferent vessels; and (3) volume of injected sclerosant. Although multiple EVs may be punctured in a single EIS session, the most prominent EV was punctured first, and the “injected sclerosant volume” was defined as the volume administered during the first puncture of the first EV.

### 2.4 EV Visibility Assessment

**FIGURE 2:**
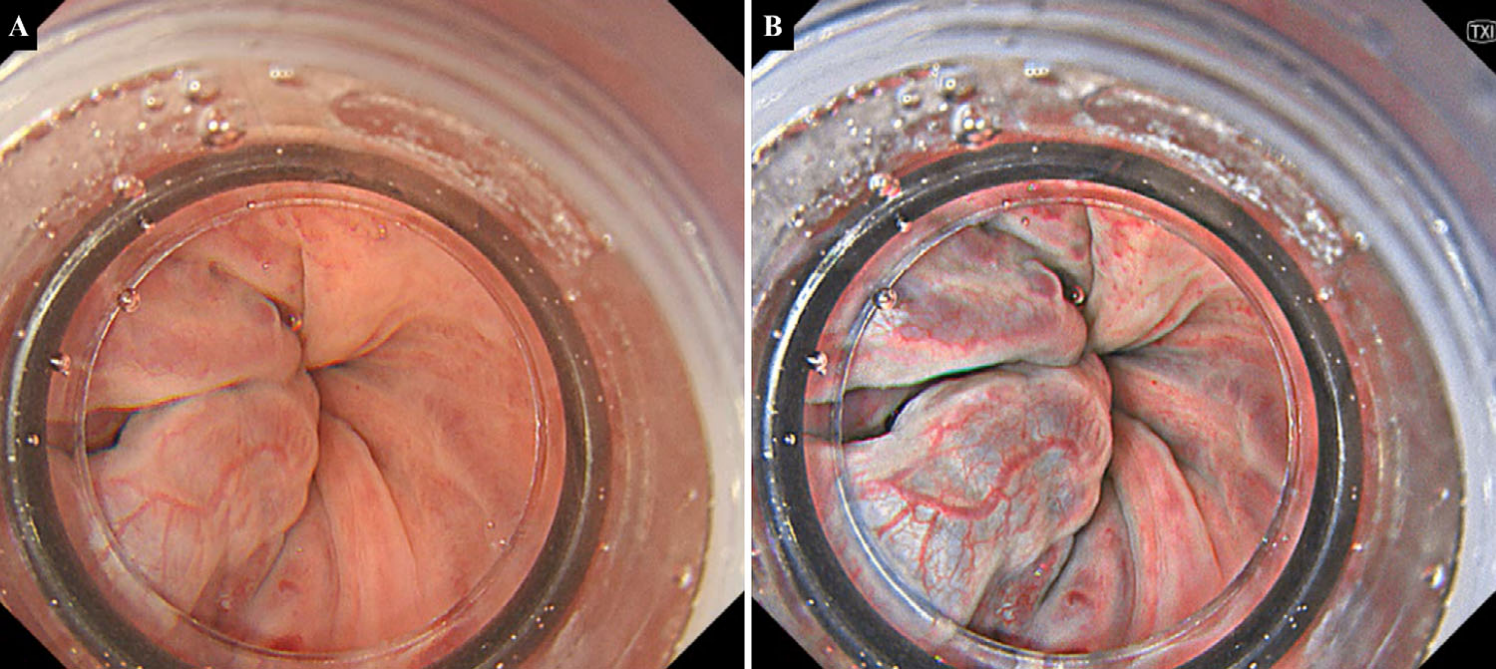
Representative endoscopic images of esophageal varices: Endoscopic images from a man in his 70s undergoing GI‐EIS with an EVL device attached to the endoscope tip. (A) WLI shows F2‐type esophageal varices in the lower thoracic esophagus. (B) Corresponding TXI image of the same area. EVL, endoscopic variceal ligation; F2, moderately enlarged varices; GI‐EIS, gel‐immersion endoscopic injection sclerotherapy; TXI, texture and color enhancement imaging; WLI, white light imaging.

The EV visibility assessment was performed on cases that underwent GI‐EIS under TXI. The analysis was conducted on the most morphologically prominent EV that was first punctured using WLI and TXI images taken from similar distances and angles (Figures 2A,B), as shown below. One of the two reviewers (Tsunetaka Kato and Takumi Yanagita) who performed EIS on several enrolled patients performed the subject visibility assessment using a five‐point scale (5, markedly superior to WLI; 4, slightly superior to WLI; 3, comparable to WLI; 2, slightly inferior to WLI; and 1, markedly inferior to WLI).

**FIGURE 3:**
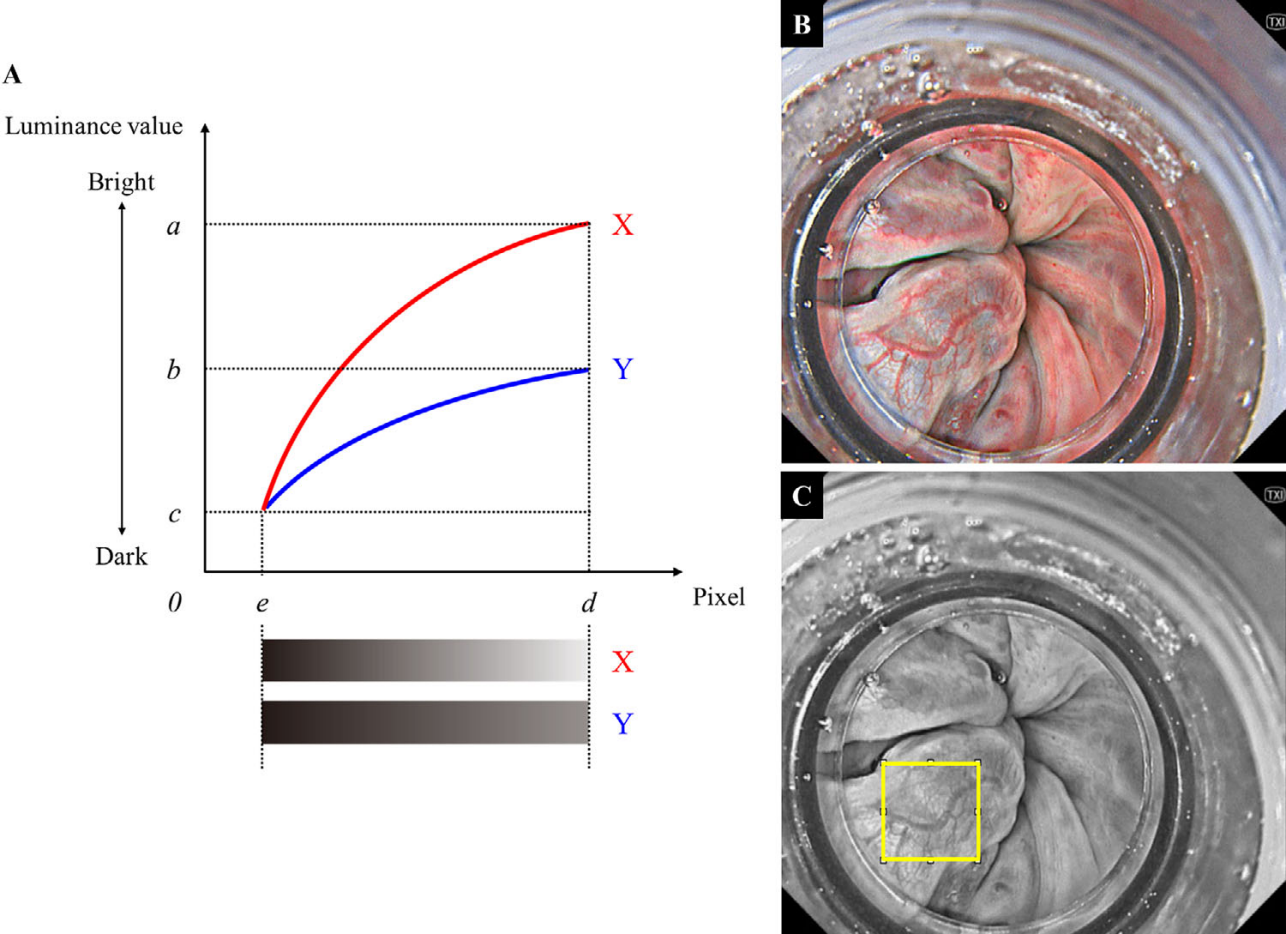
Luminance gradient analysis and calculation method: (A) Schematic illustrating the concept of the luminance gradient. The gradient was calculated as the rate of change in luminance between two points in an analyzed area. Larger gradients were considered more three‐dimensional. (B) WLI and TXI images capturing the puncture site during GI‐EIS were imported into image analysis software. (C) Imported endoscopic images to be analyzed were preprocessed by converting them to 8‐bit grayscale, and then a 100 × 100‐pixel ROI, including the puncture site, was defined. The luminance values were then calculated. EIS, endoscopic injection sclerotherapy; ROI, region of interest; TXI, texture and color enhancement imaging; WLI, white light imaging.

Using luminance gradient analysis to indirectly and quantitatively evaluate human perception of three‐dimensionality in two‐dimensional images, objective visibility was assessed [22, 23]. The luminance gradient represents the rate of change in brightness across a defined region. A smooth surface exhibits a small luminance gradient, whereas a surface with pronounced irregularities demonstrates a larger gradient. The luminance gradient of curved line X from point e to d is approximated as (a−c)/(d−e); similarly, the gradient of line Y is approximated as (b−c)/(d−e), where a > b > c and d > e (Figure 3A). Therefore, line X is perceived as more three‐dimensional than line Y. Still, WLI and TXI images before variceal puncture were imported into Fiji (National Institutes of Health, Bethesda, MD, USA; distributed by Adobe Systems) (Figure 3B). The imported endoscopic images to be analyzed were preprocessed by converting them to 8‐bit grayscale to eliminate color information, and then a 100 × 100‐pixel region of interest (ROI) containing the actual puncture site was selected (Figure 3C). The ROI can be set to any pixel size according to the endoscopic image quality, but this time, it was fixed at 100 × 100 pixels to include the puncture site. The luminance gradient was calculated as follows: (maximum luminance−minimum luminance)/141.4 (i.e., the diagonal length in pixels).

### 2.5 Statistical Analysis

Numerical data were presented as medians and interquartile ranges (IQRs). The comparison of patient backgrounds between the GI‐EIS under TXI and conventional EIS groups was evaluated using the Mann–Whitney U and Chi‐square tests and an m × n contingency table. The comparison of clinical outcomes between the GI‐EIS under TXI and conventional EIS groups was evaluated using the Mann–Whitney U and Chi‐square tests. The Wilcoxon signed‐rank test was used to compare the luminance gradient, maximum, minimum, and mean luminance values between WLI and TXI. Pearson's correlation coefficient was used for assessing the correlations between the WLI and TXI values. A *p*‐value of <0.05 was considered statistically significant. Analyses were performed using Statistical Package for the Social Sciences (version 21; IBM Corp., Armonk, NY, USA); missing WLI or TXI data were not imputed based on missing at random.

## 3 RESULTS

### 3.1 Patient and Lesion Characteristics

Data from patients who underwent GI‐EIS under TXI (*n* = 16) and conventional EIS (*n* = 16) were analyzed. The clinical characteristics and endoscopic findings are summarized in Table 1. In the GI‐EIS under TXI, the median patient age was 68 (IQR, 57.8–73.5) years, and alcohol‐related liver disease was the most common cause of cirrhosis. Regarding variceal morphology, the F2 and F3 types accounted for 81.2% and 18.8% of the cases, respectively. One patient had recurrent EVs following prior endoscopic treatment. There were no differences in patient background between the two groups.

**TABLE 1:** Comparison of patient background data between the group that underwent gel‐immersion endoscopic injection sclerotherapy under texture and color enhancement imaging (TXI) and the group that underwent conventional endoscopic injection sclerotherapy.

Characteristics	GI‐EIS under TXI (*n* = 16)	Conventional EIS (*n* = 16)	*p*‐Value^*^
Age (years), median (IQR)	68 (57.8–73.5)	68.5 (59–71.5)	0.83^*^
Sex, *n*			0.28^†^
Male	9	6	
Female	7	10	
Etiology of liver cirrhosis, *n*			
Alcohol‐related/MASH/Viral/Others	8/4/2/2	4/4/2/6	0.34^‡^
Child–Pugh classification, *n*			
A/B/C	12/4/0	7/9/0	0.07^†^
Location of varices, *n*			
Li/Lm/Ls	1/14/1	1/13/2	0.83^‡^
Form of varices, *n*			
F1/F2/F3	0/13/3	0/15/1	0.28^†^
Inner lumen of varices			
Solitary type/Complex type	14/2	15/1	0.54^†^
Color of varices, *n*			
Cw/Cb	3/13	3/13	1.00^†^
Red color sign (RC), *n*			
RC0/≥RC1	5/11	5/11	1.00^†^
Previous treatment for esophageal varices, *n* (%)	1 (6.3)	0	0.31^†^

Abbreviations: Cb, varices with blue coloration; Complex type, A varix is formed by fusion of multiple small vessels; Cw, varices with white coloration; EIS, endoscopic injection sclerotherapy; F1, straight and small‐caliber varices; F2, moderately enlarged and beady varices; F3, markedly enlarged, nodular or tumor‐shaped varices; IQR, interquartile range; Li, lower esophagus; Lm, middle esophagus; Ls, upper esophagus; MASH, metabolic dysfunction‐associated steatohepatitis; RC0, absence of red color sign; RC1, presence of red color sign; Solitary type, A varix is formed by a single large vessel; TXI, texture and color enhancement imaging.

* *p*‐Value was calculated by the Mann‐Whitney U test.

^†^ *p*‐Values are calculated by the Chi‐square test.

^‡^ *p*‐Value was calculated by an m × n contingency table.

### 3.2 Treatment Outcomes

The success rate of intravariceal sclerosant injection was comparable between GI‐EIS under TXI and conventional EIS (93.8% vs. 87.5%, *p* = 0.54) (Table 2). However, injection into the afferent vessels was significantly more successful with GI‐EIS under TXI (87.5% vs. 43.8%, *p* < 0.01). Median injected sclerosant volume was 7.8 mL. In one case, both injections failed, leading to only the paravariceal injection. In another case, sclerosant extravasation occurred before injection into the afferent vessels.

**TABLE 2:** Comparison of the clinical outcomes between gel‐immersion endoscopic injection sclerotherapy under texture and color enhancement imaging (TXI) and conventional endoscopic injection sclerotherapy.

Parameters	GI‐EIS under TXI (*n* = 16)	Conventional EIS (*n* = 16)	*p*‐Value
Success rate of intravariceal sclerosant injection, *n* (%)	15 (93.8)	14 (87.5)	0.54^§^
Success rate of sclerosant injection to the afferent vessels, *n* (%)	14 (87.5)	7 (43.8)	<0.01^§^
Injected sclerosant volume^*^ (mL), median (IQR)	7.8 (5–12)	4.5 (2.4–12.4)	0.25^**^
Puncture needle size, *n*			
23 gauge/25 gauge	15/1	15/1	1.00^§^
Gel volume used, *n*			
<200/200–400/≥400 mL	1/14/1	N/A	
Visibility score of varices^†^,^‡^ (points), median (IQR)	5 (5–5)	N/A	
Adverse events, *n* (%)	4 (25.0)	5 (31.3)	0.69^§^
Hematuria, *n*	4	3	
Increased ascites, *n*	0	2	

Abbreviations: IQR, interquartile range; N/A, not applicable; TXI, texture and color enhancement imaging.

* Injected sclerosant volume refers to the amount administered during the initial puncture

^†^ Visibility score definitions: 5 = markedly superior to white light imaging (WLI), 4 = slightly superior, 3 = comparable, 2 = slightly inferior, ^1^ = markedly inferior

^‡^ Visibility score was evaluated in 15 patients, as one patient did not have an appropriate image stored to evaluate the visibility score

^§^ *p*‐Values are calculated by the Chi‐square test.

** *p*‐Value was calculated by the Mann‐Whitney U test.

The EIS session was terminated when variceal puncture was unachievable or when the total volume of 5% EO exceeded 0.4 mL/kg body weight. EIS was repeated when residual EVs requiring further treatment were revealed during follow‐up endoscopy at 1 week later. When no such lesions were identified, paravariceal EIS with polidocanol was performed, followed by argon plasma coagulation the following week. In the GI‐EIS under TXI, the final endoscopic evaluation at 2 months following the last treatment revealed that 15 patients (93.8%) demonstrated complete EV eradication, and one patient (6.3%) experienced a reduction to F1 morphology. Adverse events comprised hematuria in three cases, suspected to be related to EO; however, cases of gel aspiration and renal dysfunction were not noted.

### 3.3 Visibility Assessment Results

Visibility assessment was performed in 15 patients, as one patient did not have an adequate image stored for evaluation (Table S1). The subjective visibility scores assigned by the EIS operators were uniformly five points in all cases, indicating that TXI provided superior visibility than WLI (Table 2).

Regarding the objective assessment, the median luminance gradient was significantly higher under TXI than under WLI (0.95 vs. 0.68; *p* < 0.01). Furthermore, the maximum and mean luminance values were higher under TXI (maximum, 230.0 vs. 202.0, *p* < 0.01; mean, 160.9 vs. 155.4, *p* = 0.01). Contrarily, the minimum luminance value was higher under WLI (101.0 vs. 5.0; *p* < 0.01) (Table 3). A positive correlation was observed between WLI and TXI for the luminance gradient and luminance values (Figure S1).

**TABLE 3:** Comparison of luminance gradient and luminance values between WLI and texture and color enhancement imaging (TXI) within the ROI (*n* = 15).

Parameters	WLI	TXI	*p*‐Value^‡^
Luminance gradient^*^, median (IQR)	0.68 (0.58–0.78)	0.95 (0.90–1.12)	<0.01
Maximum luminance value (ROI^†^), median (IQR)	202.0 (185.0–210.3)	230.0 (225.0–237.5)	<0.01
Minimum luminance value (ROI^†^), median (IQR)	101.0 (85.0–114.5)	85.0 (72.5–100.5)	<0.01
Mean luminance value (ROI^†^), median (IQR)	155.4 (145.6–163.2)	160.9 (148.8–167.4)	0.01

Abbreviations: IQR, interquartile range; ROI, region of interest; TXI, texture and color enhancement imaging; WLI, white light imaging.

* Luminance values are expressed in arbitrary pixel intensity units.

^†^ ROI standardized to 100 × 100 pixels.

^‡^ *p*‐Values calculated using the Wilcoxon signed‐rank test.

## 4 DISCUSSION

This study is the first to report the outcomes of GI‐EIS under TXI in EV patients undergoing primary prophylactic EIS. Dual evaluation of EV morphology visibility during GI‐EIS using subjective operator‐based visibility scores and objective image‐based luminance gradient analysis represents a key strength of this study.

In this study, the outcomes of GI‐EIS under TXI were favorable, with success rates of 93.8% and 87.5% for intravariceal sclerosant injection and injection into the afferent vessels, respectively. The success rate of intravariceal sclerosant injection was comparable to that of conventional EIS, but injection into the afferent vessels was more successful with GI‐EIS under TXI. This may be due to the fact that GI‐EIS with TXI enabled stable injection of the sclerosing agent with a stable field of view for a long time, and that TXI observation enabled the determination of a more suitable puncture point. Previous studies have reported variable success rates under conventional WLI with gas insufflation. Furuichi et al. reported intravariceal injection success rates of 46.2%–53.8% under such conditions [24, 25], whereas Sugawara et al. reported a 94.4% clinical success rate and an 88.2% afferent vessel injection success rate with GI‐EIS under WLI [16]. Our results of GI‐EIS under TXI were favorable compared with previous reports of standard EIS under gas insufflation and comparable to those of GI‐EIS under WLI. Furthermore, no adverse event directly associated with TXI use or gel aspiration related to GI‐EIS was observed, indicating that the procedure is effective and safe.

Regarding visibility, all endoscopists consistently rated the EV morphology as highly visible during GI‐EIS under TXI, which was further supported by objective analysis using luminance gradients and luminance values. Although luminance gradient analysis has been applied when evaluating visibility using other imaging modalities, including computed tomography, magnetic resonance imaging, mammography, and chest radiography [26], our study is the first to apply this analysis to endoscopic images. Our results revealed that TXI yielded significantly higher luminance gradients than WLI, suggesting enhanced recognition of surface irregularities under TXI. When measuring the luminance gradients, the endoscopic images were converted to grayscale, and color information was removed, which can be interpreted as improving the structural visibility when observed with TXI. Moreover, TXI generated higher maximum and mean luminance values and lower minimum luminance values than WLI. We also found a positive correlation between WLI and TXI in terms of both luminance gradient and luminance value. These findings indicate that TXI enhances the image's bright and dark components, thereby improving the overall image contrast and visibility. Overall, these subjective and objective evaluations confirmed the effectiveness of TXI in enhancing variceal visibility during GI‐EIS. The reason for selecting luminance gradient rather than color difference for evaluating images that contribute to the ease of puncture in EIS is explained below. EV puncture using EIS is performed by recognizing three‐dimensional structural differences, including height and shape, rather than two‐dimensional color differences on a flat surface. Therefore, in our study, we considered luminance gradient to be more appropriate than color difference, as it allows for an objective evaluation of structural differences.

Although only a few studies focusing on lesion detection and margin delineation have reported the usefulness of TXI for esophageal neoplasia [27, 28], its role in EV diagnosis or management has not been examined. Red dichromatic imaging (RDI), another image‐enhanced endoscopy modality, has demonstrated promise in EV management, with reports of improved variceal visibility and increased success rates of EIS under RDI [24, 25, 29, 30, 31]. Although EIS under RDI has exhibited superiority to WLI regarding treatment success, no study on TXI utility in this context has been conducted. GI‐EIS under TXI may be a valuable alternative image‐enhanced approach for EV therapy. In the present study, all included cases exhibited EVs with a morphology of ≥F2. In every case, both the subjective visibility scores and luminance gradients were higher under TXI than in WLI. Although the treatment outcomes were comparable to those of GI‐EIS under WLI, the improved visibility of GI‐EIS under TXI supports its recommendation. Furthermore, in cases where the treatment of EVs with morphology closer to F1 is required, TXI may offer greater benefits by directly contributing to improved therapeutic outcomes. Therefore, we suggest that GI‐EIS be performed under TXI, rather than under WLI, for EVs.

This study had several limitations. First, it was a single‐center retrospective analysis with a limited sample size. Second, treatment outcomes and patient characteristics were evaluated only between GI‐EIS under TXI and conventional EIS, and no direct comparisons were made with GI‐EIS under WLI or other image‐enhanced modalities, including RDI. Thus, to confirm these findings, future multicenter prospective randomized controlled trials will be necessary. Third, the luminance gradient analysis was conducted on the stored still WLI and TXI images acquired at similar, but not necessarily identical, angles or distances. Fourth, GI‐EIS under TXI and conventional EIS were performed by the same endoscopists, but because the treatment timing differs, the results may contribute to the endoscopist's skill improvement over time. Finally, the ROI selected for the gradient measurement was limited to a localized area near the puncture site and did not encompass the entire variceal field, potentially underestimating broader visibility differences.

In conclusion, GI‐EIS under TXI achieved favorable treatment outcomes and subjectively and objectively enhanced EV morphology visibility. TXI combined with gel‐immersion techniques may have contributed to the improved accuracy and efficacy of EIS for EVs.

## Ethics Statement

*Approval of the research protocol by an Institutional Review Board*: This study was conducted in accordance with the principles stipulated in the Declaration of Helsinki and was approved by the Ethics Committee of Fukushima Medical University (approval number: 2406).

## Consent

The requirement for obtaining informed consent from the patients was waived, and an opportunity to opt out was provided to them on the basis of the Ethical Guidelines for Medical and Biological Research Involving Human Subjects (https://www.mhlw.go.jp/content/001457376.pdf).

## Conflicts of Interest

The authors declare no conflicts of interest.

## Clinical Trial Registration

N/A.

## Supporting information




**TABLE S1**: Details of each GI‐EIS treatment case (*n* = 16).

**FIGURE S1**: Correlation between WLI and TXI in the luminance analysis. (A) Correlation in the luminance gradient (ρ = 0.71, 95% CI: 0.32–0.90, *p* < 0.01). (B) Correlation in the maximum luminance value (ρ = 0.71, 95% CI: 0.32–0.90, *p* < 0.01). (C) Correlation in the minimum luminance value (ρ = 0.92, 95% CI: 0.77–0.97, *p* < 0.01). (D) Correlation in the mean luminance value (ρ = 0.94, 95% CI: 0.82–0.98, *p* < 0.01). CI, confidence interval; ROI, region of interest; TXI, texture and color enhancement imaging; WLI, white light imaging.
