# Supplementary material for: Effectiveness of Gel‐immersion Endoscopic Injection Sclerotherapy Under Texture and Color Enhancement Imaging for Esophageal Varices: A Comparison of Variceal Visibility Under Gel With White Light Imaging
Source: DEN Open. 2025 Sep 22;6(1):e70201. doi: 10.1002/deo2.70201 (PMC12452998; doi:10.1002/deo2.70201)
Supplement: Supplementary file 1 — TABLE S1: Details of each GI‐EIS treatment case (n = 16). [file DEO2-6-e70201-s001.docx]

# Supplemental Table 1. Details of each GI-EIS treatment case (n = 16)

| Case | Age (years) | Sex | Cause of liver cirrhosis | Child–Pugh | EV form before treatment* | Sclerosant volume† (mL) | Sclerosant injection to the afferent vessels | Esophageal shunt by EVIS | Treatment duration‡ (min) | Adverse events | Paravariceal EIS/  Consolidation after intravariceal EIS§ | EV form after treatment |
| --- | --- | --- | --- | --- | --- | --- | --- | --- | --- | --- | --- | --- |
| 1 | 71 | M | Hepatitis B | B | F2 | 5 | Success | Absent | 30 | None | Yes | F0 |
| 2 | 77 | F | MASH | A | F2 | 0 (Failure) | Failure | Absent | 39 | None | Yes | F0 |
| 3 | 73 | M | Alcohol-related | A | F3 | 7 | Success | Absent | 40 | None | Yes | F0 |
| 4 | 51 | F | Alcohol-related | A | F2 | 5.5 | Success | Absent | 30 | None | Yes | F0 |
| 5 | 50 | F | Alcohol-related | A | F2 | 5 | Success | Absent | 56 | None | Yes | F0 |
| 6 | 73 | M | MASH | B | F2 | 16 | Success | Absent | 20 | None | Yes | F0 |
| 7 | 60 | F | AIH | A | F2 | 12 | Success | Absent | 31 | None | Yes | F0 |
| 8 | 70 | F | Hepatitis B | A | F2 | 3 | Success | Absent | 29 | None | Yes | F0 |
| 9 | 82 | F | Alcohol-related | A | F2 | 3.5 | Failure | Absent | 65 | None | Yes | F1 |
| 10 | 75 | M | Alcohol-related | A | F2 | 15 | Success | Absent | 64 | Hematuria | Yes | F0 |
| 11 | 65 | M | MASH | B | F2 | 12 | Success | Absent | 37 | Hematuria | Yes | F0 |
| 12 | 26 | M | Wilson’s Disease | B | F2 | 6 | Success | Absent | 54 | None | Yes | F0 |
| 13 | 66 | M | Alcohol-related | A | F2 | 18 | Success | Present | 21 | Hematuria | Yes | F0 |
| 14 | 75 | F | MASH | A | F3 | 11 | Success | Absent | 30 | None | Yes | F0 |
| 15 | 51 | M | Alcohol-related | A | F2 | 10 | Success | Absent | 20 | None | Yes | F0 |
| 16** | 65 | M | Alcohol-related | A | F3 | 8.5 | Success | Absent | 50 | Hematuria | Yes | F0 |

Abbreviations: GI-EIS, gel-immersion endoscopic injection sclerotherapy; M, male; F, female; EV, esophageal varix; EIS, endoscopic injection sclerotherapy; EVIS, endoscopic varicealography during injection sclerotherapy; MASH, metabolic dysfunction-associated steatohepatitis; AIH, autoimmune hepatitis; F0, complete resolution of varices; F1, straight and small-caliber varices; F2, moderately enlarged and beady varices
* Largest varix was assessed when multiple varices were present
† Sclerosant volume refers to the amount injected into the target varices
‡ Treatment duration is measured from endoscope insertion to removal
§ Follow-up treatment included additional intravariceal EIS, paravariceal EIS, or consolidation therapy with coagulation as needed

** Case 16 was not analyzed for luminance gradient and luminance values in white light imaging and texture and color enhancement imaging owing to the lack of proper image storage
