# Supplementary material for: Effectiveness of Gel‐immersion Endoscopic Injection Sclerotherapy Under Texture and Color Enhancement Imaging for Esophageal Varices: A Comparison of Variceal Visibility Under Gel With White Light Imaging
Source: DEN Open. 2025 Sep 22;6(1):e70201. doi: 10.1002/deo2.70201 (PMC12452998; doi:10.1002/deo2.70201)
Supplement: Supplementary file 2 — FIGURE S1: Correlation between WLI and TXI in the luminance analysis. (A) Correlation in the luminance gradient (ρ = 0.71, 95% CI: 0.32–0.90, p < 0.01). (B) Correlation in the maximum luminance value (ρ = 0.71, 95% CI: 0.32–0.90, p < 0.01). (C) Correlation in the minimum luminance value (ρ = 0.92, 95% CI: 0.77–0.97, p < 0.01). (D) Correlation in the mean luminance value (ρ = 0.94, 95% CI: 0.82–0.98, p < 0.01). CI, confidence interval; ROI, region of interest; TXI, texture and color enhancement imaging; WLI, white light imaging. [file DEO2-6-e70201-s002.pptx]

## Slide 1
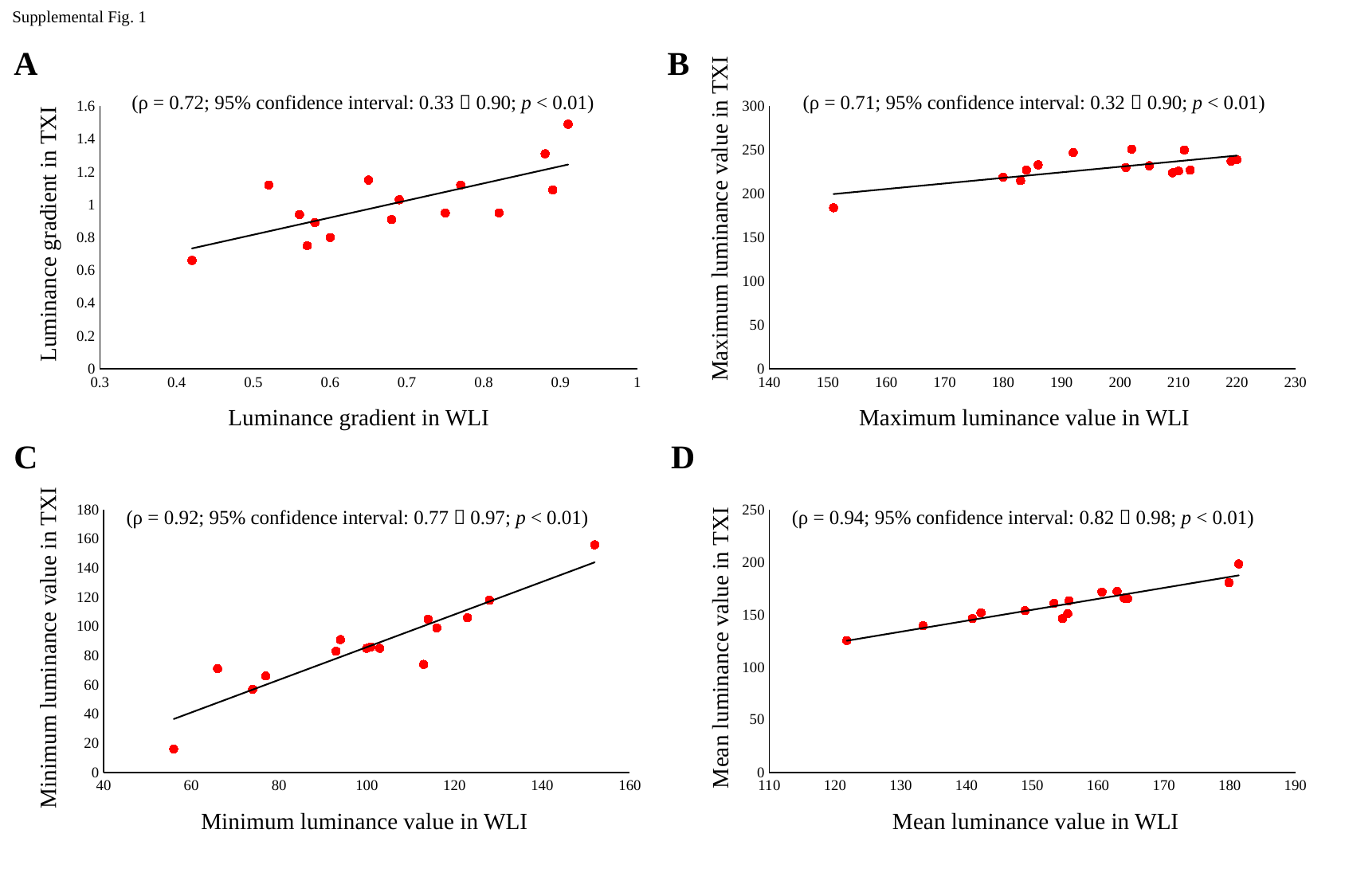

Supplemental Fig. 1
A
B
(ρ = 0.72; 95% confidence interval: 0.33－0.90; p < 0.01)
(ρ = 0.71; 95% confidence interval: 0.32－0.90; p < 0.01)
### Chart
| Category | TXI輝度勾配 |
|---|---|
### Chart
| Category | TXI max |
|---|---|Maximum luminance value in TXI
Luminance gradient in TXI
Luminance gradient in WLI
Maximum luminance value in WLI
C
D
### Chart
| Category | TXI min |
|---|---|
### Chart
| Category | TXI 平均 |
|---|---|(ρ = 0.92; 95% confidence interval: 0.77－0.97; p < 0.01)
(ρ = 0.94; 95% confidence interval: 0.82－0.98; p < 0.01)
Mean luminance value in TXI
Minimum luminance value in TXI
Mean luminance value in WLI
Minimum luminance value in WLI
